# Screening of Yeast in Various Vineyard Soil and Study on Its Flavor Compounds from Brewing Grape Wine

**DOI:** 10.3390/molecules27020512

**Published:** 2022-01-14

**Authors:** Xuzeng Wang, Zhaogai Wang, Tao Feng

**Affiliations:** 1 Agricultural and Sideline Products Processing Research Center, Henan Academy of Agricultural Sciences, Zhengzhou 450002, China; xuzeng2022@126.com (X.W.); zgwang1999@126.com (Z.W.); 2 School of Perfume and Aroma Technology, Shanghai Institute of Technology, Shanghai 200000, China

**Keywords:** *Saccharomyces cerevisiae*, isolation and purification, growth curve determination, tolerance analysis, fermentation, aroma analysis

## Abstract

In order to screen out *Saccharomyces cerevisiae* suitable for table grape fermentation, and compare it with commercial *Saccharomyces cerevisiae* in terms of fermentation performance and aroma producing substances, differences of fermentation flavor caused by different strains were discussed. In this experiment, yeast was isolated and purified from vineyard soil, 26s rDNA identification and fermentation substrate tolerance analysis were carried out, and the causes of flavor differences of wine were analyzed from three aspects: GC-MS, PCA and sensory evaluation. The results showed that strain S1 had the highest floral aroma fraction, corresponding to its high production of ethyl octanoate and other substances, and it had the characteristics of high sugar tolerance. The fruit sensory score of S3 wine was the highest among the six wines. Through exploration and analysis, it was found that compared with commercial *Saccharomyces cerevisiae*, the screened strains had more advantages in fermenting table grapes. The flavor of each wine was directly related to the growth characteristics and tolerance of its strains.

## 1. Introduction

With the improvement of people’s living standards, wine has gradually been widely accepted in China. Out of the concept of green brewing and healthy drinking, more and more people begin to make wine at home. Due to the high price of wine grapes, table grapes are mostly used as raw materials for home winemaking. At present, there are few kinds of *Saccharomyces cerevisiae* for table grape fermentation. Researchers have done a lot of work for the screening of *Saccharomyces cerevisiae*. Yeast Y03 was screened from the dough, grape skin and wine yeast, which can remove the beany-flavor effectively, by Q Tang et al. [1]. L Tao [2] had found that the relatively suitable strain for grape wine from vineyard soil was by its chemical-physical properties, and the result of the sensory evaluation was better than that of commercial yeast. A.A. Brooks [3] isolated different yeast strains from ripe banana peels to produce ethanol; five strains showed enhanced performance and were subsequently identified and assessed for important ethanol fermentation attributes, such as ethanol-producing ability, ethanol tolerance, flocculence, thermo- and osmo-tolerance.

The above research has made outstanding contributions in the field of yeast screening, however, there are few reports on the screening of *Saccharomyces cerevisiae* for table grape fermentation. In the park, where table grapes have been planted for many years, a variety of natural yeasts are born with it. After long-term natural selection and evolution, a batch of natural dominant yeasts suitable for table grape fermentation has been gradually formed, from which they can be screened as a table grape wine yeast with excellent quality [4], which is very important and has a unique style of *Saccharomyces cerevisiae* [5]. In grape cultivation and brewing in Europe, where wine has a long history, especially for infamous wine-producing areas, superior natural yeast groups are still used for fermentation. China’s major wineries attach great importance to the breeding of their own yeast, such as 7318 and 7448 yeast selected by the Yantai Changyu brewing company [6]. Xinjiang Light Industry Design Institute Professor Huang Zonghang [7] and others used Dushan red grape to separate and screen wine yeast and ferment the Dushan red grape to brew the famous “Loulan” ancient wine. These yeasts laid the foundation for the formation of wine characteristics and brands [8].

China’s table grape production has been the first in the world for many years, but the table grape processing industry is relatively backward, and the research on winemaking is less important. In addition to the thin skin, hard meat, low juice yield and low sugar content of table grapes, a lacking of yeast for table grape fermentation [9] are also one of the important reasons for the poor aroma and flavor of table grape wine. Nonetheless, the selection of *Saccharomyces cerevisiae* to produce an excellent aroma is particularly necessary and meaningful. Through experiments and exploration, four strains of *Saccharomyces cerevisiae* were screened from the vineyard soil, and then the aroma components of table grape wine were analyzed. Combined with the comparison of commercial *Saccharomyces cerevisiae* wine, the reasons for the differences of aroma components of wine caused by different strains were analyzed, so as to accumulate some experience and lay a foundation for carrying out this research work in the future.

## 2. Results and Analysis

### 2.1. Isolation and Screening of Saccharomyces Cerevisiae

**Figure 1:**
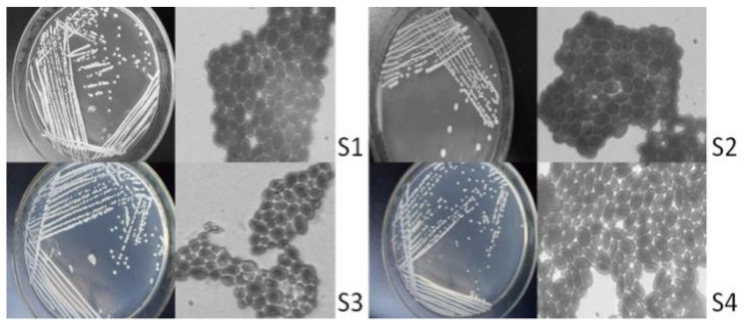
Cell morphology and colony morphology. Note: the magnification of the microscope is 1000 times.

There were 16 strains of bacteria isolated and purified from vineyard soil samples, including 3 strains of *Mould*, 3 strains of *Actinomycesbovis*, and 6 strains of *C. tropicalis*. Through strain observation and microscopic examination, 4 strains were preliminarily isolated as yeast strains (Figure 1), hereinafter referred to as S1, S2, S3 and S4.

As seen from the isolation results (Figure 1), the yeast colonies are round and milky white, among which the surfaces of S2, S3 and S4 are not smooth surfaces. The reproductive mode of cells is budding. S1 and S3 strains have round and oval forms; S2 and S4 strains have an oval shape.

### 2.2. Identification Results of Four Strains

**Figure 2:**
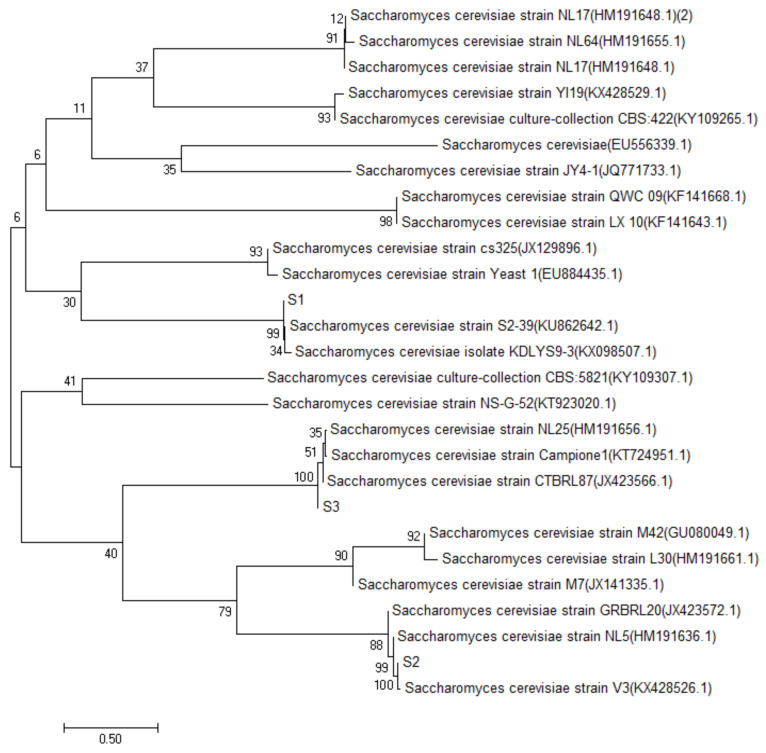
S1, S2, S3 yeast phylogenetic tree.

**Figure 3:**
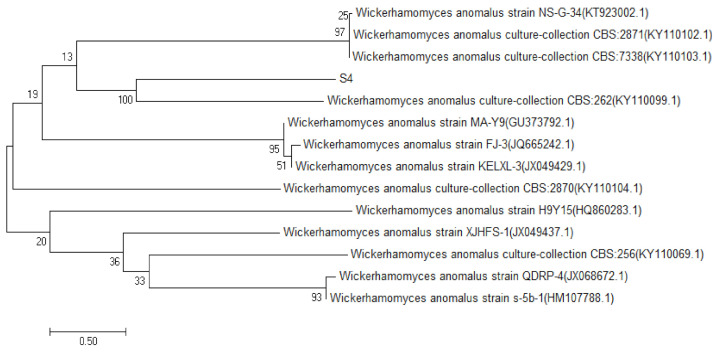
S4 yeast phylogenetic tree.

The sequencing data of S1, S2, S3 and S4 yeast strains were searched and matched by BLAST search in the GenBank database, by the establishment of the phylogenetic tree [10]. The results of S1, S2 and S3 are shown in Figure 2. The similarity between S1 and the *Saccharomyces cerevisiae* strain S2–39, was 99%, and the same was true for S2 and *Saccharomyces cerevisiae* strain NL5; S3 and *Saccharomyces cerevisiae* strain CTBRL87 had a high homology similarity (100%). The results of strain S4 are shown in Figure 3, which had a high homology similarity (100%) to abnormal *Wickham* yeast (*wickerhamomycesanomalus* culture collection CBS: 262). According to the above comparison results, S1, S2 and S3 were identified as *Saccharomyces cerevisiae* and S4 as the heterologous yeast, *Wickerhamomyces anomalus*.

### 2.3. Determination of Yeast Growth Curve

**Figure 4:**
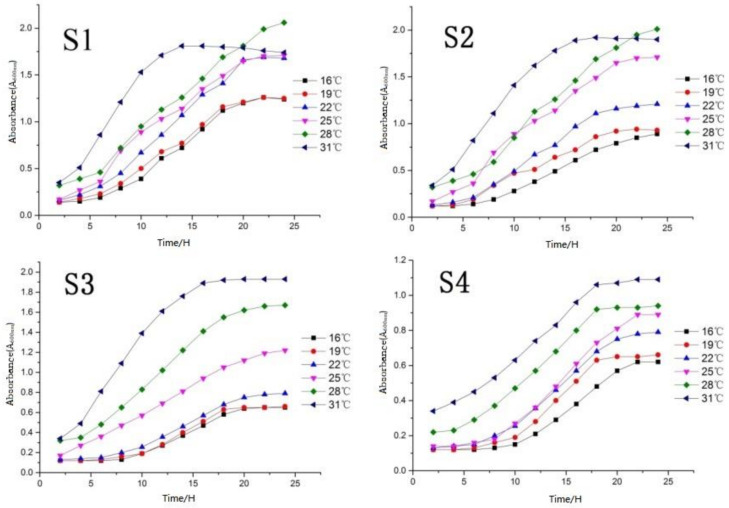
Determination of optimum growth temperature of yeast.

According to the yeast growth temperature curve (Figure 4), the four yeasts showed physiological characteristics of slow growth in the range of 16–22 °C, especially S3 and S4. This is far lower than the value (A_600nm_) of another two yeasts (S2 was 1.25, and S1 was 1.5) at the same temperature closed to 22 °C. This indicates the low temperature (lower than 22 °C) reproductive ability of S3 and S4 yeasts was poor, and there may be the possibility of infection due to weak vitality in growth. The growth status of S2 and S3 changed obviously at 25 °C by the value (A_600nm_) increase of 0.5 (S2) and 0.4 (S3). Furthermore, S1 and S4 at the growth period (25 °C) changed little, indicating that 25 °C is the critical temperature for the vigorous and weak growth of the four yeasts.

Four screened strains all showed strong vitality under the temperature of 28 °C. In especial, S1 and S2 were still in the logarithmic growth period with a rapid growth rate (25 h), indicating that this temperature (28 °C) is very suitable for the growth of S1 and S2 yeasts and can be used as the optimum growth temperature of the two strains.

S1 and S2 had obviously entered the decline period after growing to 15 h, indicating that S1 and S2 yeast cannot adapt to a high-temperature (exceed 30 °C) environment; S3 and S4 yeast entered the logarithmic phase (30 °C) longer than S1 and S2, and the value (A_600nm_) reaching the equilibrium phase was higher than the previous temperature gradient, however, they also entered the equilibrium phase in approximately 15 h. Furthermore, 31 °C can be used as the optimal growth temperature of S3 and S4.

### 2.4. Yeast Tolerance Test

#### 2.4.1. Acid-Base Tolerance Test

The pH ranges between 2.5–4.5 (acidic) on the dry red and dry white wine [11]. It can be seen in Table 1 that the growth status of the four yeasts was good in the acidity range of pH 2.0–3.5, indicating that the four selected yeasts were suitable for brewing dry red and dry white wine. Strain S4 could tolerate an acidic environment below pH 2.0 and could be identified as an acid-tolerant yeast, which indicated that wine fermented by S4 may have high acidity. Two parallel groups of the S2 strain could tolerate the alkaline growth environment of pH 12.0 and could be identified as alkaline tolerant yeast. S1 and S3 yeast could not grow in a highly acidic environment (below pH 2.0) and a high alkaline environment (above pH 12.0). When used for fermentation, the pH of the fermentation broth needs to be adjusted to the range of 2.0–3.5.

#### 2.4.2. High Glucose Tolerance Test

The sugar content in wine fermentation broth determines the wine precision after the completion of fermentation. The higher the sugar content, the higher the wine precision [12].

According to the results of the sugar tolerance test (Table 2), the growth status of the four yeasts was good under the sugar content of 0.5%, indicating that the four yeasts could normally use glucose to produce alcohol and could be used as *Saccharomyces cerevisiae*. When the sugar content reached 0.6%, strain S2 still grew normally; however, strain S4 has shown that it cannot grow, indicating that S4 was sensitive to sugar and could not adapt to the fermentation of a high sugar environment. Strains S1 and S3 could grow in the sugar environment of 0.7%, indicating that the wine of the two yeasts may have high wine precision.

#### 2.4.3. Alcohol Tolerance Test

The alcohol accuracy of wine is usually between 8–15°. According to GB/T 15038-2014, it can be called wine only if the alcohol content exceeds 7° [13]. Therefore, 7.0% ethanol content is selected as the minimum alcohol tolerance in this experiment.

Four strains grew well within the alcohol accuracy range of 12.0% (Table 3). When the alcohol content reached 13.0%, strain S4 cannot grow, indicating that its ethanol tolerance is weak compared to the other three strains. Strain S3 had two parallel experimental groups, which can tolerate 15.0% alcohol content, which is consistent with the good tolerance of S3 to high sugar in the previous experiment. This indicates that strain S3 can be used as the best choice for the production of dry red and dry white wine. S1 and S2 were able to withstand 13.0% and 14.0% alcohol precision, respectively.

### 2.5. Main Physical and Chemical Indexes of Wine

The order of alcohol degree (Table 4) from high to low was S3 > S1 > S4 > S2 > Anqi > Diboshi, corresponding to the amount of residual sugar (total sugar) content which reflected the utilization ability of yeast to sugar [14]. It can be seen from Table 4 that the total sugar content and total reducing sugar content corresponded to the degree of alcohol, indicating that the four strains had a strong ability to use sugars, and fermented thoroughly, corresponding to the results of Section 2.4.2 and Section 2.4.3.

The total acid content could determine the acid production capacity of yeast. Acids are the main flavor substances in wine and had a great influence on the color and wine flavor [15]. The total acid and total volatile acid of the six wine samples were S4 > S3 > S2 > Anqi > S1 > Diboshi. The acid production level of the strain was higher than that of commercial yeast, corresponding to the results of Section 2.4.1.

As an antioxidant and microbial inhibitor, sulfur dioxide plays an important role in wine fermentation [16]. However, excessive sulfur dioxide will destroy the nutrients in food and are harmful to the human body [17]. The total sulfur dioxide content in wine (Table 4) meets the standard of GB/T 15038-2006 (≤250 mg/L).

### 2.6. Analysis of Volatile Aroma Components of Fermented Wine

The substances detected by GC-MS are quantitatively analyzed, and the established calibration curve is shown in Table 5. The correlation coefficient R^2^ of 45 groups of standard curves was higher than 0.99, and the relative standard deviation (RSD) was less than 10%, therefore, the method met the quantitative requirements.

The volatile components of four yeast wines were analyzed by headspace solid-phase microextraction and GC-MS, using commercial yeast wine as a contrast. A total of 45 kinds of eight categories of aroma compounds were detected (Table 6), including 15 kinds of esters, 12 kinds of alcohols, 5 kinds of terpenes, 5 kinds of aldehydes, 4 kinds of acids, 3 kinds of ketones and 2 kinds of phenols. The common volatile compounds of six wines are ethyl acetate, ethyl butyrate, ethyl caproate, ethyl octanoate, isoamyl acetate, isobutanol, isoamyl alcohol and phenylethanol. Furthermore, 2,3-butanediol and 2-octanone can be identified as the main aroma substances in six wines, which is consistent with the research of Mayr C M et al. [18].

According to Table 6, 33 volatile compounds were detected in S1 wine, including 11 kinds of esters, 9 kinds of alcohols and 4 kinds of acids, 3 kinds of aldehydes, 3 kinds of ketones, 2 kinds of terpenoids, 1 kind of phenol—10 more than Anqi yeast wine (ethyl dodecanoate, tetradecanoic acid, ethyl acetate, ethyl hexadecanoate, acetophenone, geranyl acetone, limonene e β-Cedrene, 2-methylbutanol, 2-ethylhexanol, etc.), and more than 5 kinds of Diboshi yeast wine (isobutyl acetate, ethyl tetradecanoate, nonanal, decanal, etc.). Esters produced fruit and flower fragrances in bacterial metabolism and wine aging endow [19]. Ethyl tetradecanoate has the smell of sweet cream, and ethyl hexadecanoate has the smell of fruit and vanilla [20], while isobutyl acetate has the aroma of raw pear and raspberry [21]. Aldehydes and ketones are mainly formed by the decarboxylation of acids and oxidation of alcohols [22]; geranyl acetone has the smell of flowers and tropical fruits [23], nonanal has the smell of melon and nuts, and decanal has the smell of orange peel.

A total of 38 volatile compounds were detected in the S2 wine, which was the largest variety of substances in the six wines, including 13 kinds of esters, 10 kinds of alcohols, 4 kinds of acids, 3 kinds of aldehydes, 2 kinds of ketones, 4 kinds of terpenoids and 2 kinds of phenols–15 more than Anqi yeast wine (ethyl Dodecanoate, ethyl tetradecanoate, methyl hexadecanoate, ethyl hexadecanoate, 2-methylbutanol, 2-ethylhexanol, nonanal, geranyl) Acetone, etc.), and more than 10 kinds of Diboshi yeast wine (isobutyl acetate, ethyl tetradecanoate, methyl acetate, phenylethyl acetate, lemon alkene e β-Cedrene, 2,6-di-tert-butyl-p-cresol, etc.). The content of terpenoids and heterocyclic is low, but it is important to increase the flavor richness of wine [24], and limonene e belongs to monoterpenoids, which has a taste similar to lemon [25]. Polyphenols are the most important compound antioxidant in grapes [26], 2,6-di-tert-butyl-p-cresol has a slight camphor smell.

Compared with commercial yeast, the contents of ethyl heptanoate, isobutyl acetate, ethanol, propanol, n-hexanol and acetic acid in S3 wine were significantly higher, especially the ethanol content, ranking first among the six wines, corresponding to the results of Section 2.4.2 and Section 2.4.3; The contents of phenylethanol and acetic acid in S4 wine were significantly higher than those of commercial yeast and it was the highest among the six wines, corresponding to the results of Section 2.4.1. Combined with the above comparative analysis, it can be seen that compared with commercial yeast, screened yeasts produced unique compounds by fermentation, especially ethyl dodecanoate, ethyl tetradecanoate, ethyl hexadecanoate, 2-methylbutanol, 2-ethylhexanol, nonanal Decanal, limonene e β-cedrene, etc., in S1 and S2 wines.

### 2.7. Key Volatile Compounds OAV

The contribution of various compounds to the aroma of wine cannot be accurately identified only by the type and relative content of aroma substances; the aroma activity value (OAV) was added to further explore the key volatile aroma substances in wine. Allen et al. [27] found that the compounds with OAV value > 1 contributed significantly to the flavor of wine, and the larger the OAV, the higher the contribution to the aroma. However, though the substances with the OAV value < 1 did not have an adverse effect on the aroma, these substances often play a synergistic role.

There are 8 substances with the OAV value > 1 calculated according to the detection results of GC-MS (Table 7), which are considered as key components of wine aroma, including 6 substances with an OAV value > 10, and 4 substances with an OAV value > 100 (ethyl butyrate, ethyl caproate, ethyl octanoate, isoamyl acetate). The OAV value of ethyl octanoate is larger than 1000. These substances are considered essential aroma compounds in wine, and most of them are esters, indicating that esters are the characteristic aroma substances in wine, which is similar to the results of Styger et al. [28].

### 2.8. Principal Component Correlation Analysis of Volatile Compounds

In order to further clarify the differences of components and relationship of aroma substances in wines fermented by six yeasts, the PCA model was used and the correlation of the experimental results was analyzed.

**Figure 5:**
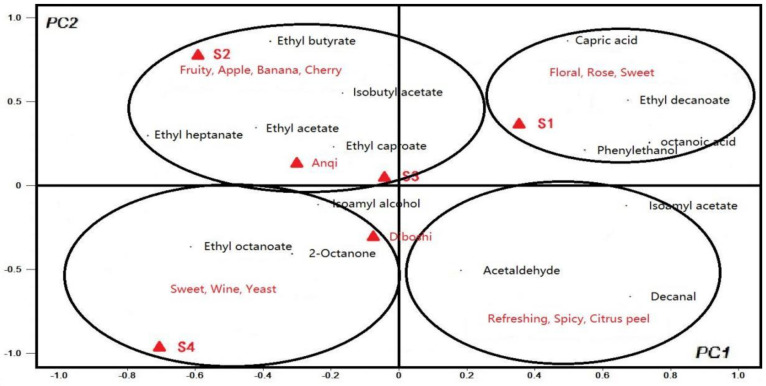
PCA of 15 volatile compounds with OAV greater than 1 to the first two principal components.

Take PC1 value as X variable and PC2 value as the y variable to generate the PCA diagram, as shown in Figure 5. In the first quadrant, aroma substances related to S1 wine are ethyl decanoate, phenylethanol, octanoic acid and capric acid. The aroma description of these substances is in line with the aroma of flowers, roses and sweet [31], corresponding to its high OAV value in S1 wine. In the second quadrant, the compounds that contribute more to S2 wine are ding ethyl acetate, ethyl heptanoate, etc. These substances have the fruity smell of grapes, bananas and cherries [32], and have a high OAV value in S2 wine. Based on PC1, S1 wine was significantly different from other wine samples. Based on PC2, S2 is obviously different from other wines sample [33], indicating that the aroma evaluation of S1 and S2 wines were the best.

The PCA results of S3 wine and Anqi yeast wine are similar, as shown in Figure 5. The aroma substances that contributed greatly to both S3 wine and Anqi yeast wine are B Ethyl acetate (grape and cherry aroma), ethyl butyrate (apple aroma) and isoamyl acetate (banana aroma), which illustrated the characteristics of the fruit aroma of the two wines.

**Figure 6:**
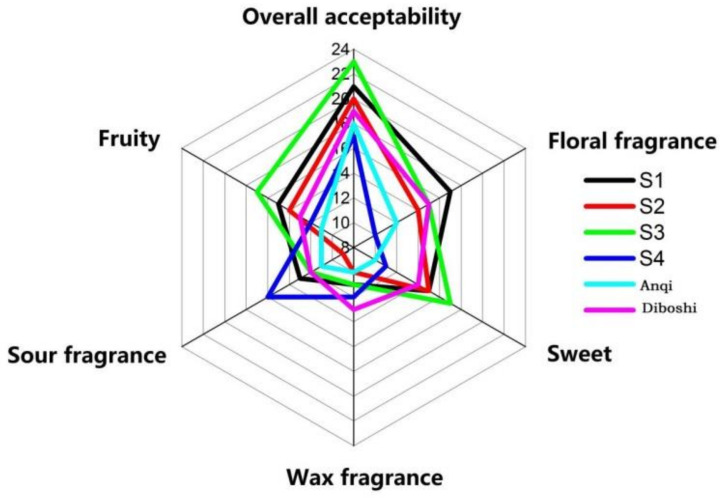
Six kinds of yeast fermentation wine aroma evaluation map.

Sensory evaluation was carried out on six yeast wines (Figure 6). The aroma evaluation of the wines of S1, S2 and S3 were generally acceptable. The sensory scores of jam (fructose flavor) and fruit trees (banana, pineapple and other fruit flavors) of S3 wine were the highest value in wines and was consistent with the ethanol produced by S3 fermentation in the results of GC/MS. Additionally, it is consistent with Styger [28] that the flavor intensity of wine is related to the type and mass concentration of alcohol compounds. When the wine reaches a certain alcohol concentration, the yeast will die. The higher the alcohol concentration, the richer the wine aroma. After the yeast dies, the cell wall breaks down and releases amino acids, fatty acids, polysaccharides, etc., which greatly affect the wine style and aroma stability. The S3 strain had high alcohol tolerance and a long survival time, so it can ferment thoroughly and provide a mellow taste. Additionally, this may be the reason for the highest sensory score of S3 wine, corresponding to the results of Section 2.4.2 and Section 2.4.3.

The sensory score of floral fragrance in S1 wine was the highest, which was consistent with the results of GC/MS. Corresponding to ethyl caproate (brandy flavor), ethyl decanoate (coconut flavor), ethyl dodecanoate (grass flavor and flower flavor) and 1-octadecene, which is consistent with Ana [34] that esters and terpenoids can enhance the fruit aroma and flower aroma in the wine.

In conclusion, the overall aroma acceptability scores of the six yeast wines were S3 > S1 > S2 > Diboshi > Anqi > S4.

## 3. Materials and Methods

### 3.1. Materials and Reagents

Fresh table grape “Zuijinxiang” was purchased in Songjiang district (Shanghai, China) in August 2016. Soil samples were collected from Fengxian, Chongming, Songjiang (Shanghai, China), Wuhan (Hubei, China), Shaoxing (Zhejiang, China), Heze (Shandong, China), Tieling (Liaoning, China), Taiyuan (Shanxi, China), Xuanhua (Hebei, China) and Suzhou (Anhui, China) vineyard soil. Commercial Saccharomyces cerevisiae was purchased from Shanghai Anqi yeast Co., Ltd. (Shanghai, China) and Shandong Yantai Diboshi brewing machine Co., Ltd. (Yantai, Shandong, China).

The yeast culture mediums PDA and YPD were purchased from Sigma-Aldrich Trading Co., Ltd. (Shanghai, China).

### 3.2. Experimental Method

#### 3.2.1. Isolation and Activation of Saccharomyces Cerevisiae in Vineyard Soil

The yeast of soil samples was obtained by enrichment and isolation [35]. The soil samples were weighed (10.0 g) and added to sterile water (200 mL) for culture (28 °C at 200 rpm for 20 min) in an incubator (Boxun Co. Ltd., Shanghai, China). The culture medium (diluted to 10^−3^, 10^−4^, 10^−5^, respectively) was evenly coated on a PDA medium plate [36], and yeast colonies were identified as Saccharomyces cerevisiae by microscope (Zhengxi Instrument Equipment Co. Ltd., Shanghai, China) were separated and cultured for 2-3 generations [37] to obtain pure yeast.

#### 3.2.2. 26S rDNA Identification

The yeast DNA was identified by DNA extraction, PCR amplification, agarose gel electrophoresis detection and sequence comparation. DNA was extracted by a GBS yeast DNA extraction kit (Jiebeisi biogenic Technology Co. Ltd., Nanjing, Jiangsu, China) with the template D1 region universal primers [38] NL1 (5’-GCATATCAATAAGCGGAGGAAAAG-3’) and NL4 (5’-GGTCCGTGTTTCAAGACGG-3 ‘).

PCR amplification was based on the 50 μL reaction system with the reaction procedure as follows: 95 °C pre-denaturation for 10 min; 94 °C denaturation for 1 min; 54 °C annealing for 1 min; 72 °C extended for 1.5 min, 36 cycles; Last step was 72 °C extended for 5 min.

The phylogenetic tree [39] (calculated by mega 7.0 software (Mega Limited, Auckland, New Zealand)) of the isolated yeast was constructed by the NJ (neighbor-joining) method based on the amplified and sequenced comparation products [40] by the NCBI database for BLAST homology sequence analysis.

#### 3.2.3. Determination of Yeast Growth Curve

The growth curve was determined according to the absorbance value by ultraviolet spectrophotometer (Meipuda Instrument Co., Ltd., Shanghai, China). Samples were taken (5 mL) every 2 h to measure the absorbance value (A_600nm_). Culture conditions of the samples were set as 16 °C, 19 °C, 22 °C, 25 °C, 28 °C, 31 °C, respectively, for 24 h (200 rpm). We repeated the measurement three times for each strain at each temperature.

### 3.3. Yeast Tolerance Test

#### 3.3.1. Acid-Base Tolerance Test

The acid-base tolerance of the strain was measured in 10 different pH growth environments as follows: pH 1.0, pH 1.5, pH 2.0, pH 2.5, pH 3.0, pH 3.5, pH 10.0, pH 11.0, pH 12.0 and pH 13.0. Four isolated yeasts were inoculated into EP tubes (5 mL) with each pH gradient for 4 days at the temperature obtained in 3.2.3. We added each yeast culture medium to a solid plate medium cultured for 48 h and observed the growth status. “YY” for good growth; “Y” for general growth; “B” for poor growth (or basically no growth). Each experiment was repeated 3 times.

#### 3.3.2. High Glucose Tolerance Test

The glucose tolerance of the strain was measured in seven growth environments with different glucose concentrations as follows: 0.2%, 0.3%, 0.4%, 0.5%, 0.6%, 0.7% and 0.8%. Four isolated yeasts were inoculated into EP tubes (5 mL) with each glucose concentration’s gradient for 4 days at the temperature obtained in 3.2.3. We added each yeast culture medium to a solid plate medium cultured for 48 h and observed the growth status. “YY” for good growth; “Y” for general growth; “B” for poor growth (or basically no growth). Each experiment was repeated 3 times.

#### 3.3.3. Alcohol Tolerance Test

The alcohol tolerance of the strain was measured in seven growth environments with different glucose concentrations as follows: 7%, 9%, 11%, 12%, 13%, 14% and 15%. Four isolated yeasts were inoculated into EP tubes (5 mL) with each alcohol concentration’s gradient for 4 days at the temperature obtained in 3.2.3. We added each yeast culture medium to a solid plate medium cultured for 48 h and observed the growth status. “YY” for good growth; “Y” for general growth; “B” for poor growth (or basically no growth). Each experiment was repeated 3 times.

### 3.4. Grape Juice Fermentation

Table grape “Zuijinxiang” was weighed (500 g) and added (washed and crushed) into a fermentation tank (glass material, Shanghai, China) with sulfur regulating tablets (SO_2_ > 99%, 200 mg/L), and the sugar content was adjusted to 25° Brix [41]. Yeast (107 CFU/mL) was added to the grape mash for fermentation at the temperature obtained in Section 3.2.3. The harvesting indication based on gravity change (close to 0.997 and remains unchanged for two consecutive days) of grape mash by hydrometer [42] (range 1.0–1.1, Tuohong Instrument Technology Co. Ltd., Guangdong, China). The basic physical and chemical indexes of wine samples were measured (total sugar content, total reducing sugar content, alcohol volume fraction, total acid content, total volatile acid content, total sulfur dioxide content) according to the GB/T 15038-2014. Each experiment was repeated 3 times.

### 3.5. Sensory Evaluation

The sensory evaluation team consisted of 12 trained personnel [43] (6 men and 6 women, aged 25–30 years) according to ISO 13300-2. The evaluation method was carried out according to Jackson’s [44] sensory environment and operation steps and quoted according to the sensory analysis method of Rutan [45]. A sensory description and evaluation table for wine aroma was established (Table 8). The sensory evaluation of wine was conducted 3 times, and the average value was taken.

### 3.6. Extraction of Volatile Components in Wine51

Volatile components in wine extracted by solid-phase microextraction [46] are as follows: wine (5 mL) was put into a headspace bottle (15 mL) for a water bath (55 °C, 30 min) with 2-Octanol (600 mg/L, 20 μL) as an internal standard and a CAR/PDMS extraction head (75 μL) was inserted (30 min).

### 3.7. GC-MS Analysis

The GC-MS analysis was carried out with an Agilent 5975 GC-MSD system (Agilent Technology, Santa Clara, CA, USA). A Hp-Innowax capillary column (60 m × 0.25 mm × 0.25 μm) was used with helium as the carrier gas (0.8 mL/min). GC oven: the initial temperature was 40 °C, kept for 2 min, raised to 230 °C at the rate of 5 °C/min, and kept for 15 min. The detector temperature was 250 °C; carrier gas was a He flow rate of 1 mL/min, with no split-flow kind. The MS condition: the four-stage rod temperature was 150 °C; the ion source temperature was 230 °C; the interface temperature was 250 °C; electron ionization source electron energy was 70 EV. The quality scanning range was 10~450 u [47].

Qualitative analysis was set as follows: all ion scanning (SCAN) mass spectrometry [48] was used for analysis with reference to the NIST11 spectrum library and compared with the RI reported in the literature, and compared with the RI and MS spectral images of the standard of the substance under the same conditions to determine a substance.

Quantitative analysis: 2-Octanol internal standard method was used for quantification. The calibration curve is drawn by the peak area ratio of the internal standard (2-Octanol) solution with different concentrations of each standard compound under the same conditions as the sample to be tested. The standard (100 μL) of each compound was put into a volumetric flask (10 mL) and fixed the volume with absolute ethanol. The dilution gradients were set at: 250 mg/L, 100 mg/L, 50 mg/L, 10 mg/L and 1 mg/L, respectively. Each experiment was repeated 3 times.

(1) $${\mathrm{RI}}_{x}=\left(\frac{\mathrm{lg}\left(t_{\left(x\right)}\right)-\;\mathrm{lg}\left(t_{\left(z\right)}\right)}{\mathrm{lg}\left(t_{\left(z\;+1\right)}\right)-\mathrm{lg}\left(t_{\left(z\right)}\right)}+\;z\right)\times 10$$

where t_(x)_ is the retention time of volatile substances; t_(z)_ is the retention time of n-alkanes with the same carbon atom as the volatile substance; z is the number of carbon atoms of volatile substances.

(2) $${\mathrm{RI}}_{x}=\left(\frac{\mathrm{lg}\left(t_{\left(x\right)}\right)-\;\mathrm{lg}\left(t_{\left(z\right)}\right)}{\mathrm{lg}\left(t_{\left(z\;+1\right)}\right)-\mathrm{lg}\left(t_{\left(z\right)}\right)}+\;z\right)\times 10$$

where w_i_ is the concentration of volatile substances, mg/kg; m_S_ is the content of the internal standard, μg; A_i_ is the peak area of volatile substances; A_s_ is the internal standard peak area of substance; m_0_ is the quality of wine sample, g.

### 3.8. Data Analysis

The key aroma components of wine were evaluated by measuring the aroma activity value (OAV) of aroma substances. OAV is an important indicator of specific compound sample odors, whose value is equal to the ratio of compound concentration to an olfactory threshold in the corresponding solute [49]. The threshold values of all aroma compounds were determined in alcohol solution according to GB/T 33406-2016 [50].

The correlation between OAV > 1 compounds and yeast wine was analyzed. PCA analyzis was carried out by unscrambler 9.7, and its data relevance was processed by centralization and standardization (1/SDEV). The significant difference level was taken as *p* < 0.05 [51]. The model was cross-validation correct.

## 4. Conclusions

Four strains of yeast were successfully isolated from vineyard soils in Chongming District (Shanghai, China), Shaoxing (Zhejiang, China), Heze (Shandong, China) and Wuhan (Hubei, China). The results showed that S1, S2 and S3 were Saccharomyces cerevisiae, while S4 was an abnormal Wickham’s yeast. The temperature growth curves of the four selected strains were measured and drawn. The optimum growth temperature of S1 (A_600nm_ = 2.06) and S2 (A_600nm_ = 2.01) was 28 °C, and the optimum growth temperature of S3 (A_600nm_ = 1.93) and S4 (A_600nm_ = 1.09) was 31 °C. In the yeast tolerance test, strain S1 had a good high glucose tolerance (0.7%), high ethanol content tolerance (13.0%) and sulfur dioxide tolerance (650 mg/L); strain S2 had good alkali tolerance (pH 12.0) and high glucose tolerance (0.6%); and strain S3 had good high glucose tolerance (0.7%), ethanol tolerance (15%) and osmotic pressure tolerance (sodium chloride concentration 1.3%). Strain S4 has a good acid resistance (pH 1.0) and sulfur dioxide tolerance (650 mg/L).

Table grape “Zuijinxiang” was fermented with four isolated strains, and Anqi and Diboshi yeast wine was used as the control. A total of 45 aroma components were detected by GC-MS, including 12 kinds of alcohols, 15 kinds of esters and alkenes, 5 kinds of terpenoids, 5 kinds of aldehydes, 4 kinds of acids, 4 kinds of ketones and 2 kinds of phenols. Compared with commercial yeast wine, S1 wine had a higher content of ethyl caproate, ethyl decanoate, ethyl dodecanoate, ethyl decanoate, phenylethanol, octanoic acid and decanoic acid. The contents of folic acetone, 2,6-di-t-butyl-p-cresol and 4-vinyl-2-methoxyphenol were high in S2 wine, and S3 wine had a high content of ethyl acetate and ethanol content, while S4 wine had a higher acetic acid content.

This experiment explored the differences between screened yeast and commercial yeast in fermenting table grape “Zuijinxiang”, and studied the reasons why different fermentation flavors were caused by different strains. This experiment also made a prospect for the direction of screening table grape yeast in the future, as well as for the genetic improvement of table grapes.

## Figures and Tables

**Table 1:** Acid and alkali tolerance results of four yeasts.

Yeast	pH Value									
	1.0	1.5	2.0	2.5	3.0	3.5	10.0	11.0	12.0	13.0
S1	B	B	Y	YY	YY	YY	Y	Y	B	B
S1	B	B	Y	YY	YY	YY	Y	Y	B	B
S1	B	B	Y	YY	YY	YY	Y	Y	B	B
S2	B	B	Y	Y	Y	YY	Y	Y	Y	B
S2	B	B	Y	Y	Y	YY	Y	Y	Y	B
S2	B	B	Y	Y	Y	YY	Y	Y	B	B
S3	B	B	YY	Y	YY	YY	Y	Y	B	B
S3	B	B	YY	Y	YY	YY	Y	Y	Y	B
S3	B	B	YY	YY	YY	YY	Y	Y	B	B
S4	Y	Y	Y	YY	YY	YY	Y	Y	B	B
S4	Y	Y	Y	YY	YY	YY	Y	Y	B	B
S4	Y	Y	Y	YY	YY	YY	Y	Y	B	B

“YY” means good growth; “Y” means average growth; “B” means no growth.

**Table 2:** Four strains of yeast for high glucose tolerance test results.

Yeast	Sugar Degree/%						
	0.2	0.3	0.4	0.5	0.6	0.7	0.8
S1	YY	YY	YY	YY	YY	Y	B
S1	YY	YY	YY	YY	YY	Y	B
S1	YY	YY	YY	YY	YY	Y	B
S2	YY	YY	YY	YY	YY	B	B
S2	YY	YY	YY	YY	YY	B	B
S2	YY	YY	YY	YY	YY	B	B
S3	YY	YY	YY	YY	Y	Y	B
S3	YY	YY	YY	YY	Y	Y	B
S3	YY	YY	YY	YY	Y	Y	B
S4	YY	YY	YY	YY	B	B	B
S4	YY	YY	YY	YY	B	B	B
S4	YY	YY	YY	YY	B	B	B

“YY” means good growth; “Y” means average growth; “B” means no growth.

**Table 3:** Four kinds of yeast test results on ethanol tolerance.

Yeast	Ethanol Concentration/%						
	7.0	9.0	11.0	12.0	13.0	14.0	15.0
S1	YY	YY	YY	YY	Y	B	B
S1	YY	YY	YY	YY	Y	B	B
S1	YY	YY	YY	YY	Y	B	B
S2	YY	YY	YY	YY	YY	Y	B
S2	YY	YY	YY	YY	YY	Y	B
S2	YY	YY	YY	YY	YY	Y	B
S3	YY	YY	YY	YY	YY	Y	Y
S3	YY	YY	YY	YY	YY	Y	Y
S3	YY	YY	YY	YY	YY	Y	B
S4	YY	YY	YY	Y	B	B	B
S4	YY	YY	YY	Y	B	B	B
S4	YY	YY	YY	Y	B	B	B

“YY” means good growth, “Y” means average growth, “B” means no growth.

**Table 4:** Physical and chemical indicators analysis results.

Indexes	Wine					
	S1	S2	S3	S4	Anqi	Diboshi
Alcohol content (%, *V*/*V*)	12.66 ± 0.21	12.15 ± 0.11	13.02 ± 0.36	12.32 ± 0.11	11.83 ± 0.23	11.42 ± 0.06
Total sugar content (g/L)	21.68 ± 0.32	25.73 ± 0.12	21.31 ± 1.04	25.07 ± 0.20	28.62 ± 0.32	29.48 ± 0.22
Total reducing sugar content (g/L)	12.33 ± 0.14	17.21 ± 0.23	11.67 ± 0.41	15.24 ± 0.31	17.38 ± 0.43	17.65 ± 0.16
Total acid content (g/L H_2_SO_4_)	4.65 ± 0.16	5.18 ± 0.32	5.32 ± 0.23	5.71 ± 0.11	4.73 ± 0.16	4.38 ± 0.33
Total volatile acid content (g/L)	0.42 ± 0.02	0.39 ± 0.10	0.34 ± 0.02	0.40 ± 0.14	0.32 ± 0.06	0.37 ± 0.02
Total sulfur dioxide content (mg/L)	23.21 ± 0.07	22.68 ± 0.31	21.32 ± 0.43	21.36 ± 0.13	20.74 ± 0.26	21.17 ± 0.14

Note: the contents of total sugar and total reducing sugar are calculated by glucose.

**Table 5:** Linear regression equation, correlation coefficient and relative standard deviation of 45 compounds.

NO.	Volatile Components	Linear Regression Equation	R^2^	RSD (%)
1	Ethyl acetate	y = 0.0037x + 0.0514	0.9996	5
2	Ethyl butyrate	y = 0.0112x + 0.0987	0.9994	4
3	Ethyl caproate	y = 0.6937x + 0.7259	0.9997	4
4	Ethyl heptanate	y = 0.0049x + 0.6975	0.9996	3
5	Ethyl octanoate	y = 0.0664x + 0.1367	0.9995	7
6	Ethyl decanoate	y = 0.0196x + 0.0055	0.9989	6
7	Isobutyl acetate	y = 0.0060x + 0.0953	0.9991	2
8	Isoamyl acetate	y = 0.1067x + 0.0006	0.9989	4
9	Ethyl Dodecanoate	y = 0.0056x + 0.0209	0.9993	4
10	Tetradecanoic acid ethyl ester	y = 0.1054x + 0.6007	0.9997	6
11	Methyl hexadecanoate	y = 0.0064x + 0.6510	0.9997	3
12	Ethyl palmitate	y = 0.0584x + 0.1367	0.9994	5
13	Acetic acid-2-methyl-Butyl ester	y = 0.0047x + 0.0105	0.9996	6
14	Phenylethyl acetate	y = 0.0093x + 0.0035	0.9992	6
15	9-decanoic acid ethyl ester	y = 0.0019x + 0.5760	0.9988	4
16	ethanol	y = 0.0067x + 0.0352	0.9990	6
17	Propanol	y = 0.0031x + 0.0008	0.9996	6
18	Propylene glycol	y = 0.0669x + 0.3047	0.9993	7
19	Glycerol	y = 0.0079x + 0.3540	0.9990	4
20	N-butanol	y = 0.0303x + 0.0034	0.9999	6
21	Isobutanol	y = 0.0037x + 0.2005	0.9992	8
22	Isoamyl alcohol	y = 0.0024x + 0.0097	0.9994	4
23	N-hexanol	y = 0.0057x + 0.3006	0.9996	8
24	Phenylethanol	y = 0.0064x + 0.1025	0.9995	7
25	2-methyl butanol	y = 0.0097x + 0.3315	0.9994	6
26	2-ethylhexanol	y = 0.0237x + 0.0957	0.9993	3
27	2,3-butanediol	y = 0.0054x + 0.7640	0.9997	6
28	acetic acid	y = 0.0634x + 0.3401	0.9997	3
29	Caproic acid	y = 0.0008x + 0.2670	0.9996	4
30	Octanoic acid	y = 0.0534x + 0.3407	0.9993	6
31	Capric acid	y = 0.0375x + 0.0240	0.9993	4
32	acetaldehyde	y = 0.0034x + 0.0490	0.9998	6
33	Nonanal	y = 0.1039x + 0.0006	0.9991	3
34	Decanal	y = 0.0005x + 0.3207	0.9997	6
35	Acetal	y = 0.0099x + 0.0345	0.9996	4
36	2-Octanone	y = 0.0300x + 0.0759	0.9995	2
37	Acetophenone	y = 0.3104x + 0.0267	0.9997	4
38	Geranyl acetone	y = 0.0094x + 0.0537	0.9992	8
39	1-octadecane	y = 0.0601x + 0.0044	0.9999	6
40	D-terpene diene	y = 0.0035x + 0.9142	0.9990	3
41	Limonene e	y = 0.0149x + 0.0008	0.9996	6
42	β-Cedrene	y = 0.0004x + 0.0070	0.9997	7
43	1-undecylene	y = 0.0402x + 0.0391	0.9990	4
44	2,6-DI-TERT-BUTYL-Hydroxy p-cresol	y = 0.0002x + 0.3307	0.9990	6
45	4-vinyl-2-METHOXYPHENOL	y = 0.0070x + 0.0054	0.9990	5

**Table 6:** Volatile components and content of six wines.

No.	Volatile Aroma Component	Relative to Internal Standard Content (mg/L)						KI ^a^	Identification Method ^b^
		S1	S2	S3	S4	Anqi	Diboshi		
	Esters								
1	Ethyl acetate	1.493	2.340	5.633	0.877	6.317	2.621	601	MS,RI
2	Ethyl butyrate	0.691	1.396	0.491	0.201	0.389	0.287	793	MS,RI
3	Ethyl caproate	0.495	0.341	0.088	0.156	1.037	1.259	990	MS,RI
4	Ethyl heptanate	0.537	0.762	0.088	-^c^	0.030	0.025	1080	MS,RI
5	Ethyl octanoate	3.771	2.239	0.205	0.243	2.602	4.949	1184	MS,RI
6	Ethyl decanoate	3.593	2.825	-	0.010	0.314	0.910	1380	MS,RI
7	Isobutyl acetate	0.106	0.191	0.581	-	0.224	-	764	MS,RI
8	Isoamyl acetate	5.756	4.212	3.737	0.766	3.886	3.404	866	MS,RI
9	Ethyl Dodecanoate	2.151	0.879	-	-	-	0.548	1578	MS,RI
10	Tetradecanoic acid ethyl ester	0.096	0.251	0.078	-	-	-	1778	MS,RI
11	Methyl hexadecanoate	-	0.598	-	-	-	-	1909	MS,RI
12	Ethyl palmitate	0.232	0.784	0.275	-	-	0.099	1978	MS,RI
13	Acetic acid-2-methyl Butyl ester	-	-	0.092	0.240	-	-	869	MS,RI
14	Phenylethyl acetate	-	0.190	-	0.096	0.185	-	1224	MS,RI
15	9-decanoic acid ethyl ester	-	-	-	-	-	0.808	1978	MS,RI
	alcohols								
16	ethanol	26.826	52.744	62.579	23.329	40.510	51.980	440	MS,RI
17	Propanol	1.410	-	4.138	-	2.179	1.036	574	MS,RI
18	Propylene glycol	-	-	-	-	-	0.105	1605	MS,RI
19	Glycerol	-	0.155	-	-	-	-	2300	MS,RI
20	N-butanol	0.871	0.795	0.478	-	0.493	0.680	654	MS,RI
21	Isobutanol	1.989	2.727	1.119	0.599	0.178	0.875	607	MS,RI
22	Isoamyl alcohol	5.607	4.587	4.197	2.770	10.097	5.799	730	MS,RI
23	N-hexanol	-	0.073	0.054	-	0.012	0.059	832	MS,RI
24	Phenylethanol	2.250	1.686	0.617	0.700	0.439	0.539	1121	MS,RI
25	2-methyl butanol	0.477	0.287	-	0.690	-	-	869	MS,RI
26	2-ethylhexanol	0.125	0.989	-	-	-	0.875	1030	MS,RI
27	2,3-butanediol	0.993	0.185	0.117	0.350	0.128	0.926	1556	MS,RI
	Acids								
28	acetic acid	1.230	0.878	3.594	9.203	0.523	0.912	625	MS,RI
29	Caproic acid	0.230	0.037	-	-	0.082	0.054	975	MS,RI
30	Octanoic acid	0.954	0.715	0.156	-	0.067	0.220	1175	MS,RI
31	Capric acid	0.022	0.572	-	-	-	0.013	1203	MS,RI
	aldehyde								
32	acetaldehyde	0.117	0.052	-	-	-	0.276	363	MS,RI
33	Nonanal	0.047	0.061	0.011	-	-	-	1101	MS,RI
34	Decanal	0.099	-	-	-	-	-	1206	MS,RI
35	Acetal	-	0.190	-	-	-	0.407	866	MS,RI
	Ketones								
36	2-Octanone	0.673	0.444	0.194	0.813	1.819	1.329	994	MS,RI
37	Acetophenone	0.038	-	-	-	-	-	1038	MS,RI
38	Geranyl acetone	0.010	0.049	-	-	-	-	1452	MS,RI
	terpenes								
39	1-octadecane	0.160	-	-	-	-	-	1790	MS,RI
40	D-terpene diene	0.156	0.012	-	-	0.054	0.018	1041	MS,RI
41	Limonene e	-	0.079	-	-	-	-	1029	MS,RI
42	β-Cedrene	-	0.014	-	-	-	-	1419	MS,RI
43	1-undecylene	-	0.036	-	-	-	-	1883	MS,RI
	Phenols								
44	2,6-DI-TERT-BUTYL Hydroxy p-cresol	0.6630	0.202	-	-	0.011	-	1505	MS,RI
45	4-vinyl-2- METHOXYPHENOL	-	0.054	-	-	-	-	1315	MS,RI

^a^ KI: retention index of HP-innowax column. ^b^ Identification method: Ms means comparison between the Wiley database and NIST database; RI represents the comparison with the retention index in the literature. -^c^: not detected.

**Table 7:** OAV values of volatile substances in six yeast wines (OAV > 1).

Chemical Compounds/Threshold Vales (mg/L) [29]	Aroma Description [30]	OAV Values in Six Wines					
		S1	S2	S3	S4	Anqi	Diboshi
Ethyl butyrate/0.002	Sweet fruit, apple	345.500	69.000	245.500	100.500	194.500	143.500
Ethyl caproate/0.005	Sweet pineapple, banana	99.000	68.200	17.600	31.200	207.400	251.800
Ethyl octanoate/0.002	Fruit, wine	1885.500	1119.500	102.500	121.500	1301.000	2474.500
Ethyl decanoate/0.2	Fruity, sweet apple	17.9650	14.1250		0.0500	1.5700	4.5500
Isoamyl acetate/0.03	Green ripe banana smell	191.8667	140.4000	124.5667	25.533	129.533	113.467
Octanoic acid/0.5	Soap, fat	1.908	1.430	0.312		0.134	0.440
Capric acid/0.4	Fruity, waxy soap	0.157	4.086	-	-	-	0.093
Decanal/0.01	Citrus and orange peel aromas	9.900	-	-	-	-	-

**Table 8:** Wine aroma evaluation table.

Descriptive Terms	Flavor Definition
Citrus	Grapes, grapefruit, lemon, orange
Berries	Strawberry, blackcurrant, blackberry, raspberry
Lactic acid	Yogurt, cheese, cream
Spice	Clove, licorice, fennel, black pepper
Floral fragrance	Lilac, violet, jasmine
Fruit trees	Pineapple, litchi, banana
Jam	Jam, fruit lollipop
Bad flavor	Burnt, smoke, stables

## Data Availability

All details and data can be found in the text.

## Sample Availability

Samples of the PDA and YPD medium are available from the authors.
